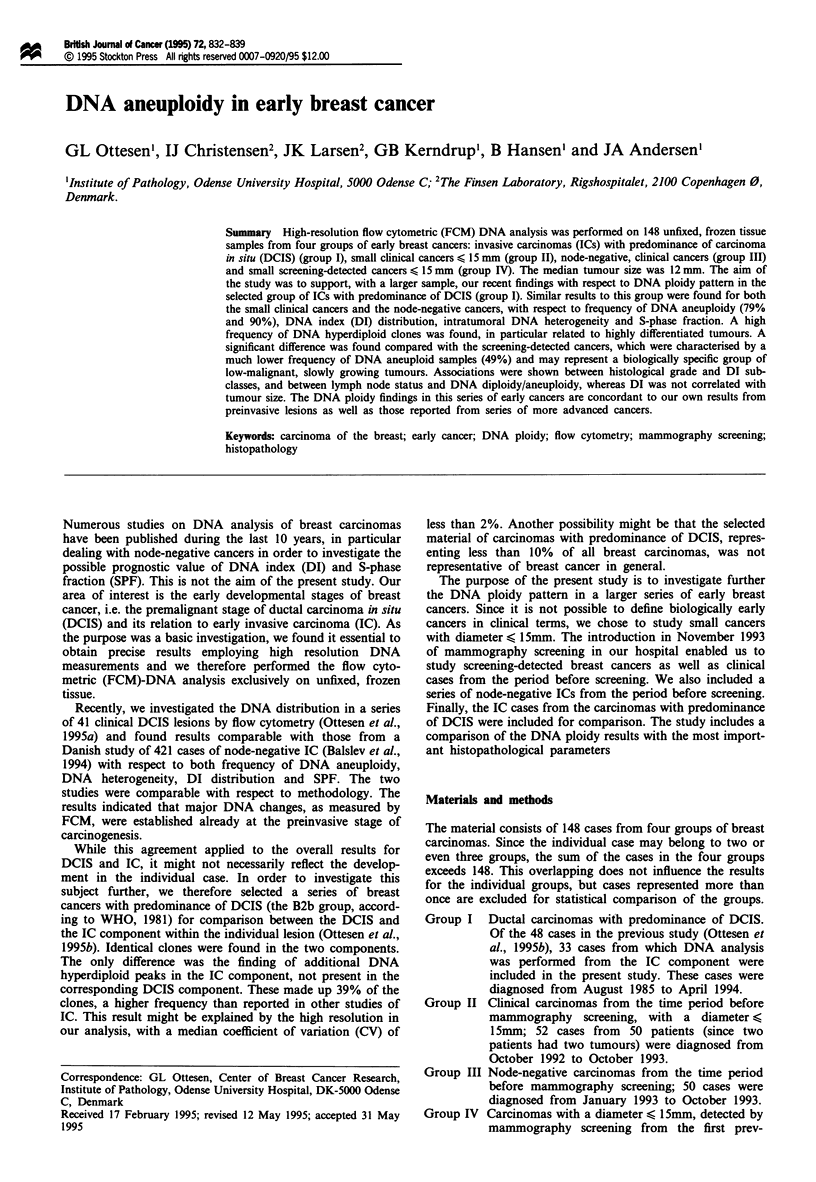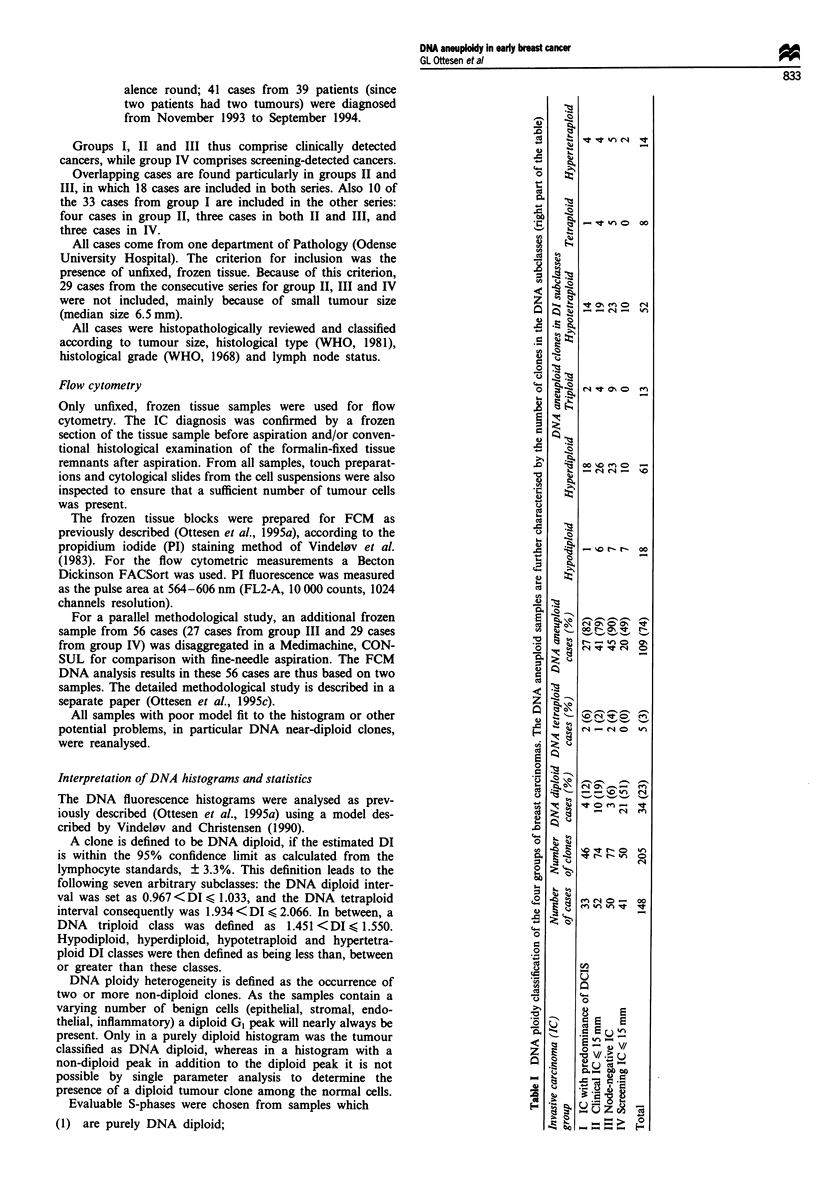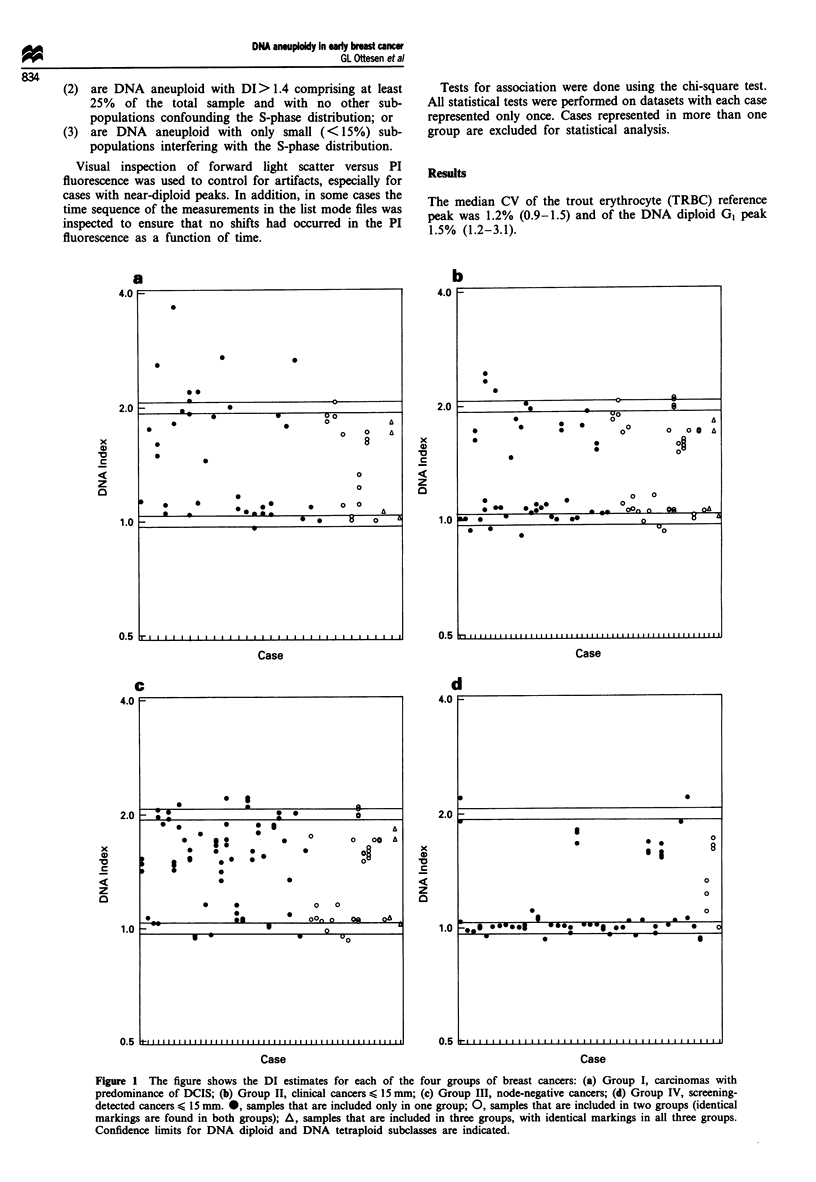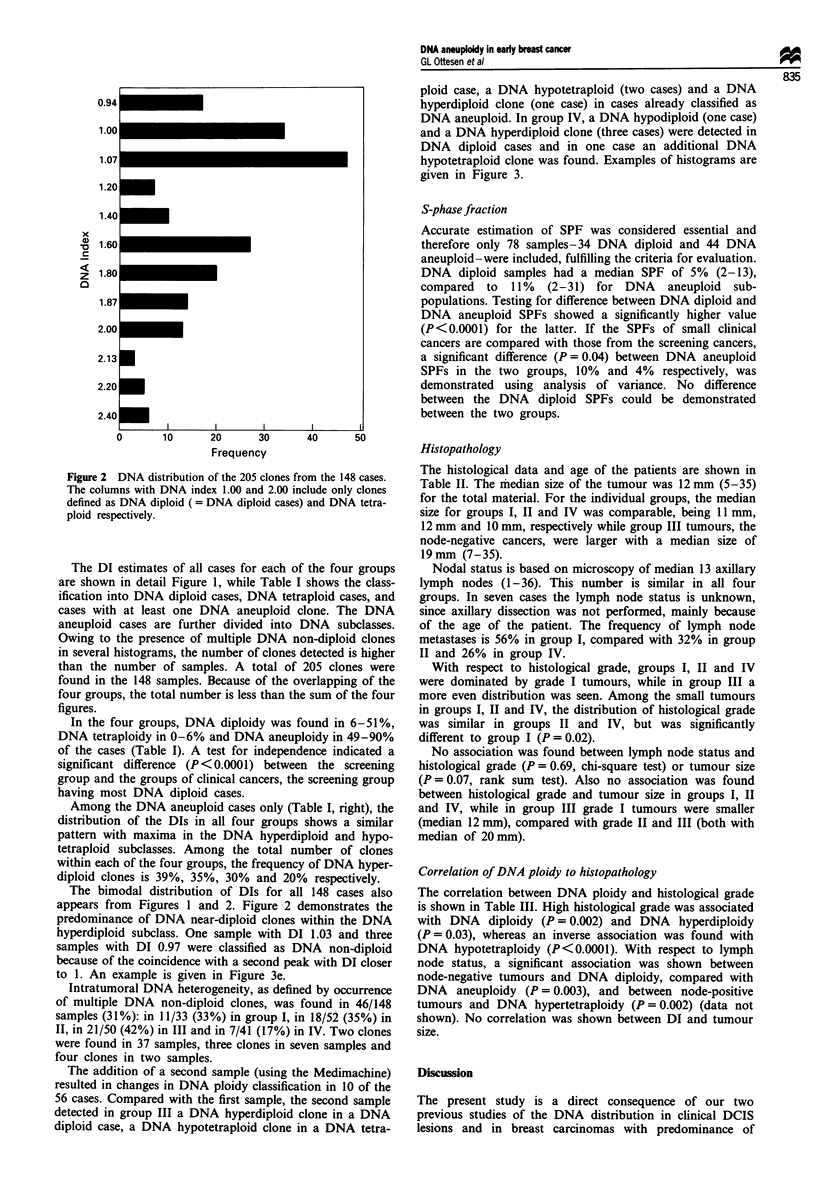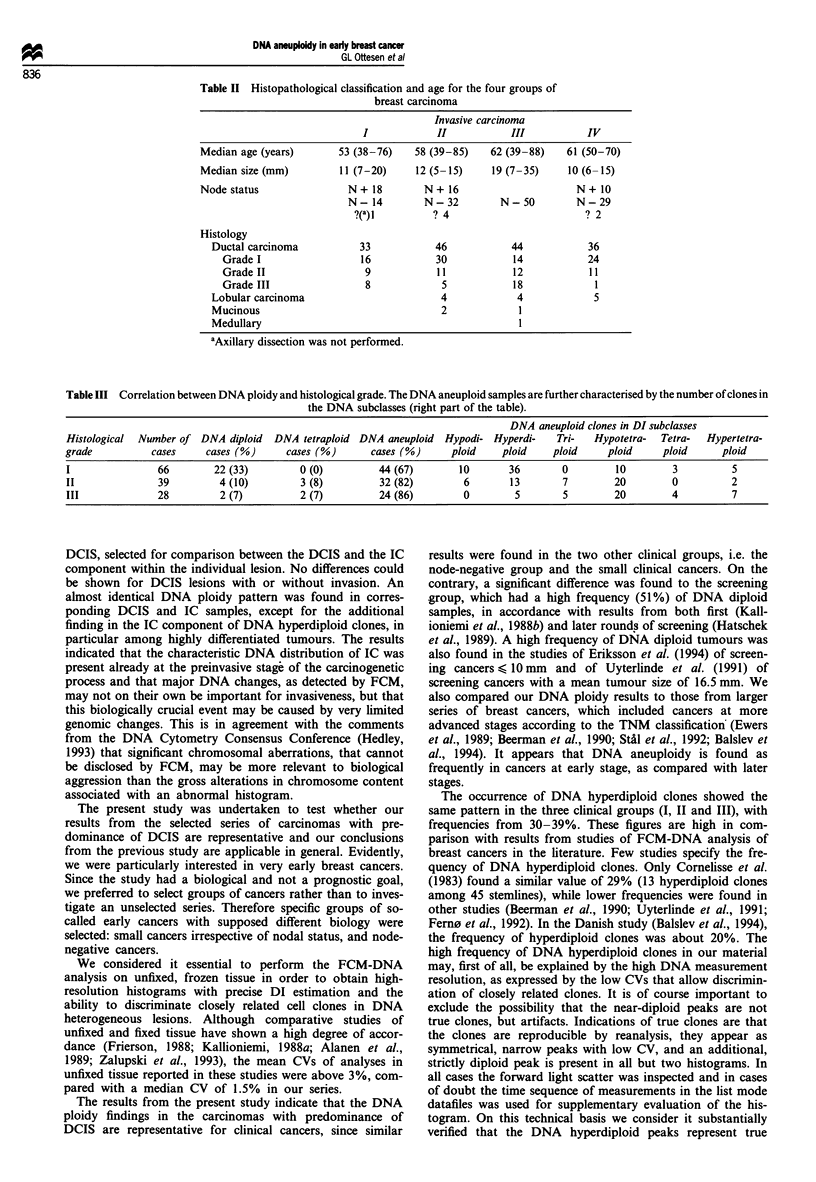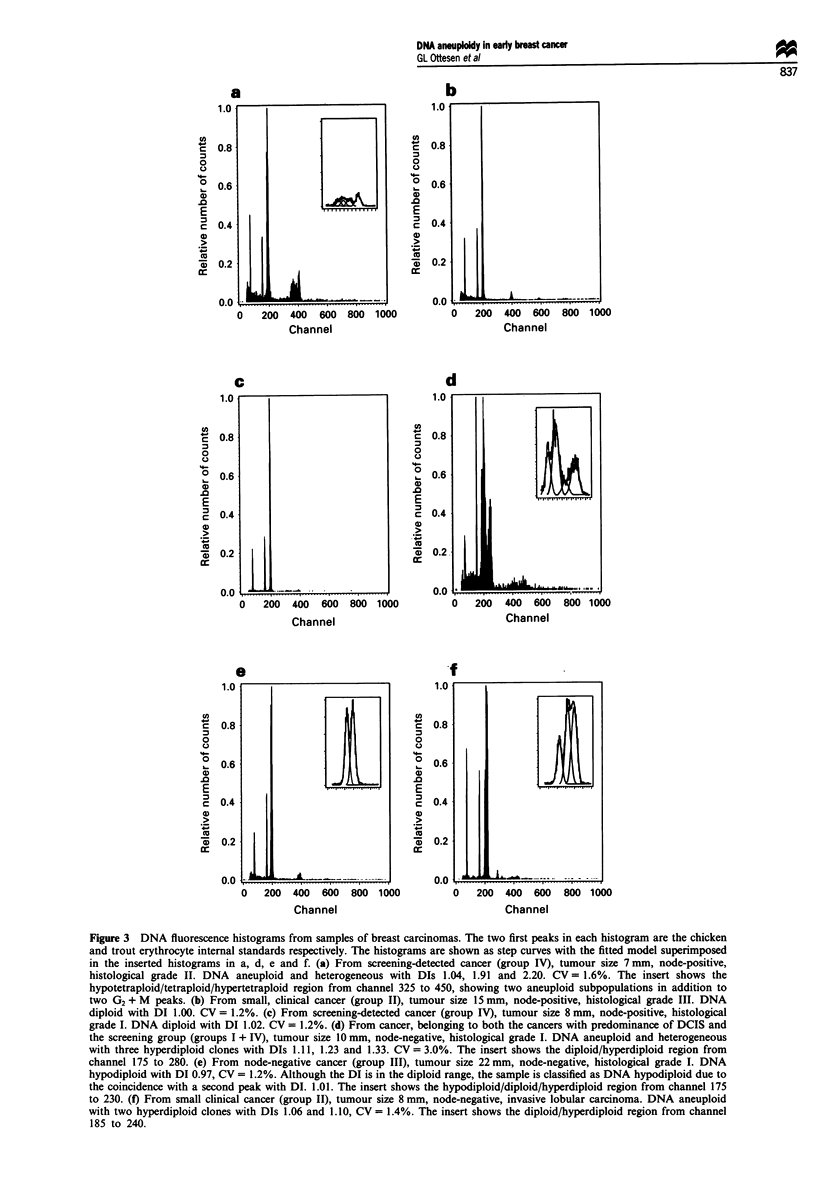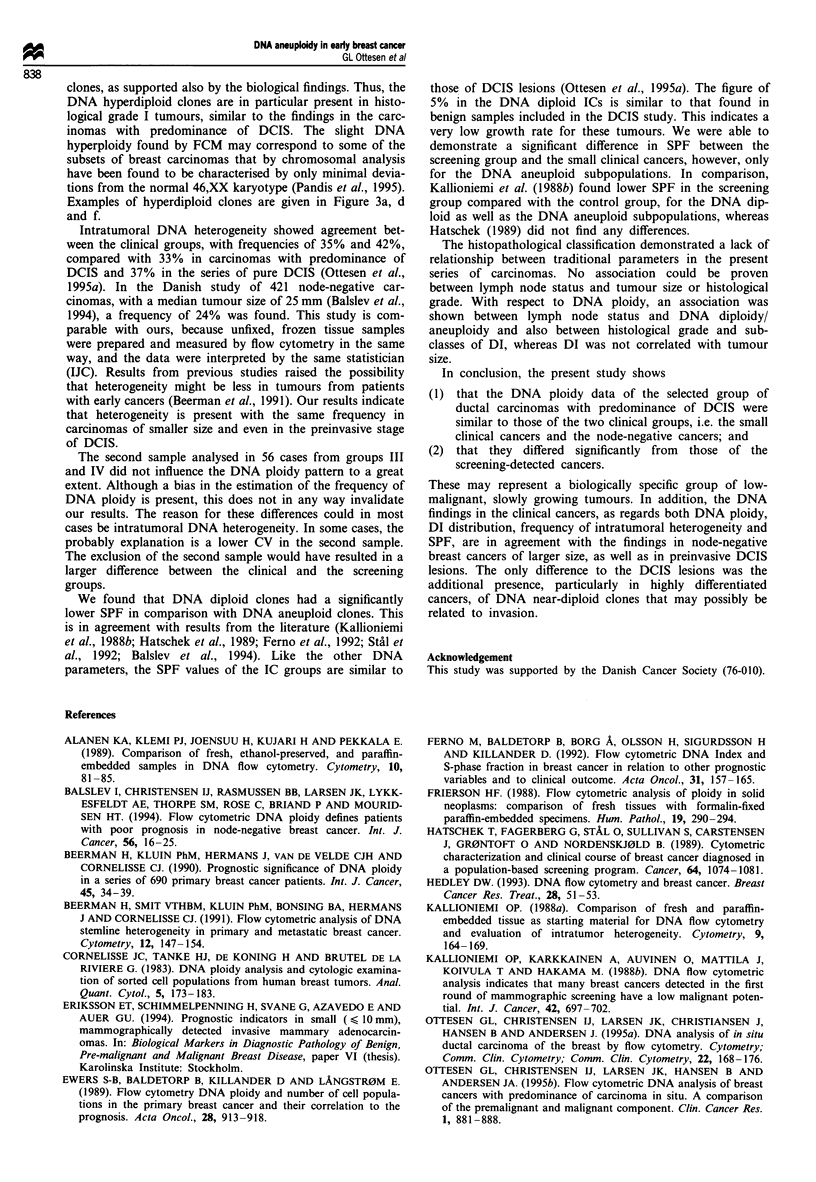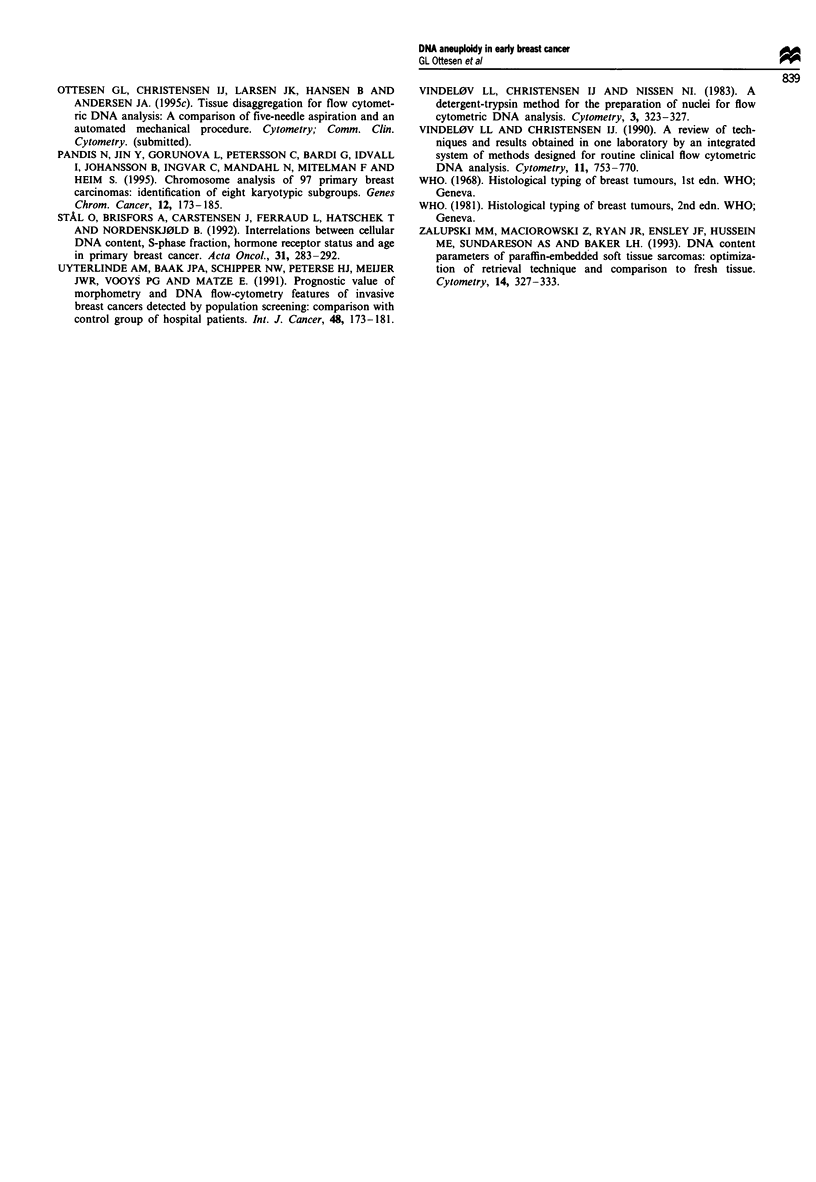# DNA aneuploidy in early breast cancer.

**DOI:** 10.1038/bjc.1995.421

**Published:** 1995-10

**Authors:** G. L. Ottesen, I. J. Christensen, J. K. Larsen, G. B. Kerndrup, B. Hansen, J. A. Andersen

**Affiliations:** Institute of Pathology, Odense University Hospital, Denmark.

## Abstract

High-resolution flow cytometric (FCM) DNA analysis was performed on 148 unfixed, frozen tissue samples from four groups of early breast cancers: invasive carcinomas (ICs) with predominance of carcinoma in situ (DCIS) (group I), small clinical cancers < or = 15 mm (group II), node-negative, clinical cancers (group III) and small screening-detected cancers < or = 15 mm (group IV). The median tumour size was 12 mm. The aim of the study was to support, with a larger sample, our recent findings with respect to DNA ploidy pattern in the selected group of ICs with predominance of DCIS (group I). Similar results to this group were found for both the small clinical cancers and the node-negative cancers, with respect to frequency of DNA aneuploidy (79% and 90%), DNA index (DI) distribution, intratumoral DNA heterogeneity and S-phase fraction. A high frequency of DNA hyperdiploid clones was found, in particular related to highly differentiated tumours. A significant difference was found compared with the screening-detected cancers, which were characterised by a much lower frequency of DNA aneuploid samples (49%) and may represent a biologically specific group of low-malignant, slowly growing tumours. Associations were shown between histological grade and DI subclasses, and between lymph node status and DNA diploidy/aneuploidy, whereas DI was not correlated with tumour size. The DNA ploidy findings in this series of early cancers are concordant to our own results from preinvasive lesions as well as those reported from series of more advanced cancers.


British Journal of Cancer (1995) 72, 832-839

© 1995 Stockton Press All rights reserved 0007-0920/95 $12.00

### DNA aneuploidy in early breast cancer

GL Ottesen[1], IJ Christensen[2], JK Larsen[2], GB Kerndrup[1], B Hansen[1] and JA Andersen[1]

[1]*Institute of Pathology, Odense University Hospital, 5000 Odense C;* [2]*The Finsen Laboratory, Rigshospitalet, 2100 Copenhagen Ø, Denmark.*

**Summary** High-resolution flow cytometric (FCM) DNA analysis was performed on 148 unfixed, frozen tissue samples from four groups of early breast cancers: invasive carcinomas (ICs) with predominance of carcinoma *in situ* (DCIS) (group I), small clinical cancers ≤ 15 mm (group II), node-negative, clinical cancers (group III) and small screening-detected cancers ≤ 15 mm (group IV). The median tumour size was 12 mm. The aim of the study was to support, with a larger sample, our recent findings with respect to DNA ploidy pattern in the selected group of ICs with predominance of DCIS (group I). Similar results to this group were found for both the small clinical cancers and the node-negative cancers, with respect to frequency of DNA aneuploidy (79% and 90%), DNA index (DI) distribution, intratumoral DNA heterogeneity and S-phase fraction. A high frequency of DNA hyperdiploid clones was found, in particular related to highly differentiated tumours. A significant difference was found compared with the screening-detected cancers, which were characterised by a much lower frequency of DNA aneuploid samples (49%) and may represent a biologically specific group of low-malignant, slowly growing tumours. Associations were shown between histological grade and DI subclasses, and between lymph node status and DNA diploidy/aneuploidy, whereas DI was not correlated with tumour size. The DNA ploidy findings in this series of early cancers are concordant to our own results from preinvasive lesions as well as those reported from series of more advanced cancers.

**Keywords:** carcinoma of the breast; early cancer; DNA ploidy; flow cytometry; mammography screening; histopathology

Numerous studies on DNA analysis of breast carcinomas have been published during the last 10 years, in particular dealing with node-negative cancers in order to investigate the possible prognostic value of DNA index (DI) and S-phase fraction (SPF). This is not the aim of the present study. Our area of interest is the early developmental stages of breast cancer, i.e. the premalignant stage of ductal carcinoma *in situ* (DCIS) and its relation to early invasive carcinoma (IC). As the purpose was a basic investigation, we found it essential to obtain precise results employing high resolution DNA measurements and we therefore performed the flow cytometric (FCM)-DNA analysis exclusively on unfixed, frozen tissue.

Recently, we investigated the DNA distribution in a series of 41 clinical DCIS lesions by flow cytometry (Ottesen *et al.*, 1995*a*) and found results comparable with those from a Danish study of 421 cases of node-negative IC (Balslev *et al.*, 1994) with respect to both frequency of DNA aneuploidy, DNA heterogeneity, DI distribution and SPF. The two studies were comparable with respect to methodology. The results indicated that major DNA changes, as measured by FCM, were established already at the preinvasive stage of carcinogenesis.

While this agreement applied to the overall results for DCIS and IC, it might not necessarily reflect the development in the individual case. In order to investigate this subject further, we therefore selected a series of breast cancers with predominance of DCIS (the B2b group, according to WHO, 1981) for comparison between the DCIS and the IC component within the individual lesion (Ottesen *et al.*, 1995*b*). Identical clones were found in the two components. The only difference was the finding of additional DNA hyperdiploid peaks in the IC component, not present in the corresponding DCIS component. These made up 39% of the clones, a higher frequency than reported in other studies of IC. This result might be explained by the high resolution in our analysis, with a median coefficient of variation (CV) of less than 2%. Another possibility might be that the selected material of carcinomas with predominance of DCIS, representing less than 10% of all breast carcinomas, was not representative of breast cancer in general.

The purpose of the present study is to investigate further the DNA ploidy pattern in a larger series of early breast cancers. Since it is not possible to define biologically early cancers in clinical terms, we chose to study small cancers with diameter ≤ 15mm. The introduction in November 1993 of mammography screening in our hospital enabled us to study screening-detected breast cancers as well as clinical cases from the period before screening. We also included a series of node-negative ICs from the period before screening. Finally, the IC cases from the carcinomas with predominance of DCIS were included for comparison. The study includes a comparison of the DNA ploidy results with the most important histopathological parameters

### Materials and methods

The material consists of 148 cases from four groups of breast carcinomas. Since the individual case may belong to two or even three groups, the sum of the cases in the four groups exceeds 148. This overlapping does not influence the results for the individual groups, but cases represented more than once are excluded for statistical comparison of the groups.

Group I Ductal carcinomas with predominance of DCIS. Of the 48 cases in the previous study (Ottesen *et al.*, 1995*b*), 33 cases from which DNA analysis was performed from the IC component were included in the present study. These cases were diagnosed from August 1985 to April 1994.

Group II Clinical carcinomas from the time period before mammography screening, with a diameter ≤ 15mm; 52 cases from 50 patients (since two patients had two tumours) were diagnosed from October 1992 to October 1993.

Group III Node-negative carcinomas from the time period before mammography screening; 50 cases were diagnosed from January 1993 to October 1993.

Group IV Carcinomas with a diameter ≤ 15mm, detected by mammography screening from the first prev-

Correspondence: GL Ottesen, Center of Breast Cancer Research, Institute of Pathology, Odense University Hospital, DK-5000 Odense C, Denmark
Received 17 February 1995; revised 12 May 1995; accepted 31 May 1995

alence round; 41 cases from 39 patients (since two patients had two tumours) were diagnosed from November 1993 to September 1994.

Groups I, II and III thus comprise clinically detected cancers, while group IV comprises screening-detected cancers.

Overlapping cases are found particularly in groups II and III, in which 18 cases are included in both series. Also 10 of the 33 cases from group I are included in the other series: four cases in group II, three cases in both II and III, and three cases in IV.

All cases come from one department of Pathology (Odense University Hospital). The criterion for inclusion was the presence of unfixed, frozen tissue. Because of this criterion, 29 cases from the consecutive series for group II, III and IV were not included, mainly because of small tumour size (median size 6.5 mm).

All cases were histopathologically reviewed and classified according to tumour size, histological type (WHO, 1981), histological grade (WHO, 1968) and lymph node status.

*Flow cytometry*

Only unfixed, frozen tissue samples were used for flow cytometry. The IC diagnosis was confirmed by a frozen section of the tissue sample before aspiration and/or conventional histological examination of the formalin-fixed tissue remnants after aspiration. From all samples, touch preparations and cytological slides from the cell suspensions were also inspected to ensure that a sufficient number of tumour cells was present.

The frozen tissue blocks were prepared for FCM as previously described (Ottesen *et al.*, 1995*a*), according to the propidium iodide (PI) staining method of Vindeløv *et al.* (1983). For the flow cytometric measurements a Becton Dickinson FACSort was used. PI fluorescence was measured as the pulse area at 564–606 nm (FL2-A, 10 000 counts, 1024 channels resolution).

For a parallel methodological study, an additional frozen sample from 56 cases (27 cases from group III and 29 cases from group IV) was disaggregated in a Medimachine, CONSUL for comparison with fine-needle aspiration. The FCM DNA analysis results in these 56 cases are thus based on two samples. The detailed methodological study is described in a separate paper (Ottesen *et al.*, 1995*c*).

All samples with poor model fit to the histogram or other potential problems, in particular DNA near-diploid clones, were reanalysed.

*Interpretation of DNA histograms and statistics*

The DNA fluorescence histograms were analysed as previously described (Ottesen *et al.*, 1995*a*) using a model described by Vindeløv and Christensen (1990).

A clone is defined to be DNA diploid, if the estimated DI is within the 95% confidence limit as calculated from the lymphocyte standards, ± 3.3%. This definition leads to the following seven arbitrary subclasses: the DNA diploid interval was set as $0.967 < DI \leqslant 1.033$, and the DNA tetraploid interval consequently was $1.934 < DI \leqslant 2.066$. In between, a DNA triploid class was defined as $1.451 < DI \leqslant 1.550$. Hypodiploid, hyperdiploid, hypotetraploid and hypertetraploid DI classes were then defined as being less than, between or greater than these classes.

DNA ploidy heterogeneity is defined as the occurrence of two or more non-diploid clones. As the samples contain a varying number of benign cells (epithelial, stromal, endothelial, inflammatory) a diploid $G_1$ peak will nearly always be present. Only in a purely diploid histogram was the tumour classified as DNA diploid, whereas in a histogram with a non-diploid peak in addition to the diploid peak it is not possible by single parameter analysis to determine the presence of a diploid tumour clone among the normal cells.

Evaluable S-phases were chosen from samples which

(1) are purely DNA diploid;

**Table I** DNA ploidy classification of the four groups of breast carcinomas. The DNA aneuploid samples are further characterised by the number of clones in the DNA subclasses (right part of the table)

| *Invasive carcinoma (IC) group* | *Number of cases* | *Number of clones* | *DNA diploid cases (%)* | *DNA tetraploid cases (%)* | *DNA aneuploid cases (%)* | *DNA aneuploid clones in DI subclasses* | | | | | |
|---|---|---|---|---|---|---|---|---|---|---|---|
| | | | | | | *Hypodiploid* | *Hyperdiploid* | *Triploid* | *Hypotetraploid* | *Tetraploid* | *Hypertetraploid* |
| I IC with predominance of DCIS | 33 | 46 | 4 (12) | 2 (6) | 27 (82) | 1 | 18 | 2 | 14 | 1 | 4 |
| II Clinical IC ⩽ 15 mm | 52 | 74 | 10 (19) | 1 (2) | 41 (79) | 6 | 26 | 4 | 19 | 4 | 4 |
| III Node-negative IC | 50 | 77 | 3 (6) | 2 (4) | 45 (90) | 7 | 23 | 9 | 23 | 5 | 5 |
| IV Screening IC ⩽ 15 mm | 41 | 50 | 21 (51) | 0 (0) | 20 (49) | 7 | 10 | 0 | 10 | 0 | 2 |
| Total | 148 | 205 | 34 (23) | 5 (3) | 109 (74) | 18 | 61 | 13 | 52 | 8 | 14 |

(2) are DNA aneuploid with DI > 1.4 comprising at least 25% of the total sample and with no other subpopulations confounding the S-phase distribution; or
(3) are DNA aneuploid with only small (<15%) subpopulations interfering with the S-phase distribution.

Visual inspection of forward light scatter versus PI fluorescence was used to control for artifacts, especially for cases with near-diploid peaks. In addition, in some cases the time sequence of the measurements in the list mode files was inspected to ensure that no shifts had occurred in the PI fluorescence as a function of time.

Tests for association were done using the chi-square test. All statistical tests were performed on datasets with each case represented only once. Cases represented in more than one group are excluded for statistical analysis.

### Results

The median CV of the trout erythrocyte (TRBC) reference peak was 1.2% (0.9–1.5) and of the DNA diploid $G_1$ peak 1.5% (1.2–3.1).

**Figure 1** The figure shows the DI estimates for each of the four groups of breast cancers: (**a**) Group I, carcinomas with predominance of DCIS; (**b**) Group II, clinical cancers ≤ 15 mm; (**c**) Group III, node-negative cancers; (**d**) Group IV, screening-detected cancers ≤ 15 mm. ●, samples that are included only in one group; O, samples that are included in two groups (identical markings are found in both groups); Δ, samples that are included in three groups, with identical markings in all three groups. Confidence limits for DNA diploid and DNA tetraploid subclasses are indicated.

**Figure 2** DNA distribution of the 205 clones from the 148 cases. The columns with DNA index 1.00 and 2.00 include only clones defined as DNA diploid ( = DNA diploid cases) and DNA tetraploid respectively.

The DI estimates of all cases for each of the four groups are shown in detail Figure 1, while Table I shows the classification into DNA diploid cases, DNA tetraploid cases, and cases with at least one DNA aneuploid clone. The DNA aneuploid cases are further divided into DNA subclasses. Owing to the presence of multiple DNA non-diploid clones in several histograms, the number of clones detected is higher than the number of samples. A total of 205 clones were found in the 148 samples. Because of the overlapping of the four groups, the total number is less than the sum of the four figures.

In the four groups, DNA diploidy was found in 6–51%, DNA tetraploidy in 0–6% and DNA aneuploidy in 49–90% of the cases (Table I). A test for independence indicated a significant difference ($P<0.0001$) between the screening group and the groups of clinical cancers, the screening group having most DNA diploid cases.

Among the DNA aneuploid cases only (Table I, right), the distribution of the DIs in all four groups shows a similar pattern with maxima in the DNA hyperdiploid and hypotetraploid subclasses. Among the total number of clones within each of the four groups, the frequency of DNA hyperdiploid clones is 39%, 35%, 30% and 20% respectively.

The bimodal distribution of DIs for all 148 cases also appears from Figures 1 and 2. Figure 2 demonstrates the predominance of DNA near-diploid clones within the DNA hyperdiploid subclass. One sample with DI 1.03 and three samples with DI 0.97 were classified as DNA non-diploid because of the coincidence with a second peak with DI closer to 1. An example is given in Figure 3e.

Intratumoral DNA heterogeneity, as defined by occurrence of multiple DNA non-diploid clones, was found in 46/148 samples (31%): in 11/33 (33%) in group I, in 18/52 (35%) in II, in 21/50 (42%) in III and in 7/41 (17%) in IV. Two clones were found in 37 samples, three clones in seven samples and four clones in two samples.

The addition of a second sample (using the Medimachine) resulted in changes in DNA ploidy classification in 10 of the 56 cases. Compared with the first sample, the second sample detected in group III a DNA hyperdiploid clone in a DNA diploid case, a DNA hypotetraploid clone in a DNA tetraploid case, a DNA hypotetraploid (two cases) and a DNA hyperdiploid clone (one case) in cases already classified as DNA aneuploid. In group IV, a DNA hypodiploid (one case) and a DNA hyperdiploid clone (three cases) were detected in DNA diploid cases and in one case an additional DNA hypotetraploid clone was found. Examples of histograms are given in Figure 3.

### *S-phase fraction*

Accurate estimation of SPF was considered essential and therefore only 78 samples–34 DNA diploid and 44 DNA aneuploid–were included, fulfilling the criteria for evaluation. DNA diploid samples had a median SPF of 5% (2–13), compared to 11% (2–31) for DNA aneuploid subpopulations. Testing for difference between DNA diploid and DNA aneuploid SPFs showed a significantly higher value ($P<0.0001$) for the latter. If the SPFs of small clinical cancers are compared with those from the screening cancers, a significant difference ($P=0.04$) between DNA aneuploid SPFs in the two groups, 10% and 4% respectively, was demonstrated using analysis of variance. No difference between the DNA diploid SPFs could be demonstrated between the two groups.

### *Histopathology*

The histological data and age of the patients are shown in Table II. The median size of the tumour was 12 mm (5–35) for the total material. For the individual groups, the median size for groups I, II and IV was comparable, being 11 mm, 12 mm and 10 mm, respectively while group III tumours, the node-negative cancers, were larger with a median size of 19 mm (7–35).

Nodal status is based on microscopy of median 13 axillary lymph nodes (1–36). This number is similar in all four groups. In seven cases the lymph node status is unknown, since axillary dissection was not performed, mainly because of the age of the patient. The frequency of lymph node metastases is 56% in group I, compared with 32% in group II and 26% in group IV.

With respect to histological grade, groups I, II and IV were dominated by grade I tumours, while in group III a more even distribution was seen. Among the small tumours in groups I, II and IV, the distribution of histological grade was similar in groups II and IV, but was significantly different to group I ($P=0.02$).

No association was found between lymph node status and histological grade ($P=0.69$, chi-square test) or tumour size ($P=0.07$, rank sum test). Also no association was found between histological grade and tumour size in groups I, II and IV, while in group III grade I tumours were smaller (median 12 mm), compared with grade II and III (both with median of 20 mm).

### *Correlation of DNA ploidy to histopathology*

The correlation between DNA ploidy and histological grade is shown in Table III. High histological grade was associated with DNA diploidy ($P=0.002$) and DNA hyperdiploidy ($P=0.03$), whereas an inverse association was found with DNA hypotetraploidy ($P<0.0001$). With respect to lymph node status, a significant association was shown between node-negative tumours and DNA diploidy, compared with DNA aneuploidy ($P=0.003$), and between node-positive tumours and DNA hypertetraploidy ($P=0.002$) (data not shown). No correlation was shown between DI and tumour size.

### Discussion

The present study is a direct consequence of our two previous studies of the DNA distribution in clinical DCIS lesions and in breast carcinomas with predominance of

**Table II** Histopathological classification and age for the four groups of breast carcinoma

| | Invasive carcinoma I | II | III | IV |
|---|---|---|---|---|
| Median age (years) | 53 (38–76) | 58 (39–85) | 62 (39–88) | 61 (50–70) |
| Median size (mm) | 11 (7–20) | 12 (5–15) | 19 (7–35) | 10 (6–15) |
| Node status | N + 18<br>N − 14<br>?([a])1 | N + 16<br>N − 32<br>? 4 | <br>N − 50 | N + 10<br>N − 29<br>? 2 |
| Histology | | | | |
| Ductal carcinoma | 33 | 46 | 44 | 36 |
| Grade I | 16 | 30 | 14 | 24 |
| Grade II | 9 | 11 | 12 | 11 |
| Grade III | 8 | 5 | 18 | 1 |
| Lobular carcinoma | | 4 | 4 | 5 |
| Mucinous | | 2 | 1 | |
| Medullary | | | 1 | |

[a]Axillary dissection was not performed.

**Table III** Correlation between DNA ploidy and histological grade. The DNA aneuploid samples are further characterised by the number of clones in the DNA subclasses (right part of the table).

| Histological grade | Number of cases | DNA diploid cases (%) | DNA tetraploid cases (%) | DNA aneuploid cases (%) | DNA aneuploid clones in DI subclasses: Hypodiploid | Hyperdiploid | Triploid | Hypotetraploid | Tetraploid | Hypertetraploid |
|---|---|---|---|---|---|---|---|---|---|---|
| I | 66 | 22 (33) | 0 (0) | 44 (67) | 10 | 36 | 0 | 10 | 3 | 5 |
| II | 39 | 4 (10) | 3 (8) | 32 (82) | 6 | 13 | 7 | 20 | 0 | 2 |
| III | 28 | 2 (7) | 2 (7) | 24 (86) | 0 | 5 | 5 | 20 | 4 | 7 |

DCIS, selected for comparison between the DCIS and the IC component within the individual lesion. No differences could be shown for DCIS lesions with or without invasion. An almost identical DNA ploidy pattern was found in corresponding DCIS and IC samples, except for the additional finding in the IC component of DNA hyperdiploid clones, in particular among highly differentiated tumours. The results indicated that the characteristic DNA distribution of IC was present already at the preinvasive stage of the carcinogenetic process and that major DNA changes, as detected by FCM, may not on their own be important for invasiveness, but that this biologically crucial event may be caused by very limited genomic changes. This is in agreement with the comments from the DNA Cytometry Consensus Conference (Hedley, 1993) that significant chromosomal aberrations, that cannot be disclosed by FCM, may be more relevant to biological aggression than the gross alterations in chromosome content associated with an abnormal histogram.

The present study was undertaken to test whether our results from the selected series of carcinomas with predominance of DCIS are representative and our conclusions from the previous study are applicable in general. Evidently, we were particularly interested in very early breast cancers. Since the study had a biological and not a prognostic goal, we preferred to select groups of cancers rather than to investigate an unselected series. Therefore specific groups of so-called early cancers with supposed different biology were selected: small cancers irrespective of nodal status, and node-negative cancers.

We considered it essential to perform the FCM-DNA analysis on unfixed, frozen tissue in order to obtain high-resolution histograms with precise DI estimation and the ability to discriminate closely related cell clones in DNA heterogeneous lesions. Although comparative studies of unfixed and fixed tissue have shown a high degree of accordance (Frierson, 1988; Kallioniemi, 1988*a*; Alanen *et al.*, 1989; Zalupski *et al.*, 1993), the mean CVs of analyses in unfixed tissue reported in these studies were above 3%, compared with a median CV of 1.5% in our series.

The results from the present study indicate that the DNA ploidy findings in the carcinomas with predominance of DCIS are representative for clinical cancers, since similar results were found in the two other clinical groups, i.e. the node-negative group and the small clinical cancers. On the contrary, a significant difference was found to the screening group, which had a high frequency (51%) of DNA diploid samples, in accordance with results from both first (Kallioniemi *et al.*, 1988*b*) and later rounds of screening (Hatschek *et al.*, 1989). A high frequency of DNA diploid tumours was also found in the studies of Eriksson *et al.* (1994) of screening cancers ⩽ 10 mm and of Uyterlinde *et al.* (1991) of screening cancers with a mean tumour size of 16.5 mm. We also compared our DNA ploidy results to those from larger series of breast cancers, which included cancers at more advanced stages according to the TNM classification (Ewers *et al.*, 1989; Beerman *et al.*, 1990; Stål *et al.*, 1992; Balslev *et al.*, 1994). It appears that DNA aneuploidy is found as frequently in cancers at early stage, as compared with later stages.

The occurrence of DNA hyperdiploid clones showed the same pattern in the three clinical groups (I, II and III), with frequencies from 30–39%. These figures are high in comparison with results from studies of FCM-DNA analysis of breast cancers in the literature. Few studies specify the frequency of DNA hyperdiploid clones. Only Cornelisse *et al.* (1983) found a similar value of 29% (13 hyperdiploid clones among 45 stemlines), while lower frequencies were found in other studies (Beerman *et al.*, 1990; Uyterlinde *et al.*, 1991; Fernø *et al.*, 1992). In the Danish study (Balslev *et al.*, 1994), the frequency of hyperdiploid clones was about 20%. The high frequency of DNA hyperdiploid clones in our material may, first of all, be explained by the high DNA measurement resolution, as expressed by the low CVs that allow discrimination of closely related clones. It is of course important to exclude the possibility that the near-diploid peaks are not true clones, but artifacts. Indications of true clones are that the clones are reproducible by reanalysis, they appear as symmetrical, narrow peaks with low CV, and an additional, strictly diploid peak is present in all but two histograms. In all cases the forward light scatter was inspected and in cases of doubt the time sequence of measurements in the list mode datafiles was used for supplementary evaluation of the histogram. On this technical basis we consider it substantially verified that the DNA hyperdiploid peaks represent true

**Figure 3** DNA fluorescence histograms from samples of breast carcinomas. The two first peaks in each histogram are the chicken and trout erythrocyte internal standards respectively. The histograms are shown as step curves with the fitted model superimposed in the inserted histograms in a, d, e and f. (**a**) From screening-detected cancer (group IV), tumour size 7 mm, node-positive, histological grade II. DNA aneuploid and heterogeneous with DIs 1.04, 1.91 and 2.20. CV = 1.6%. The insert shows the hypotetraploid/tetraploid/hypertetraploid region from channel 325 to 450, showing two aneuploid subpopulations in addition to two $G_2 + M$ peaks. (**b**) From small, clinical cancer (group II), tumour size 15 mm, node-positive, histological grade III. DNA diploid with DI 1.00. CV = 1.2%. (**c**) From screening-detected cancer (group IV), tumour size 8 mm, node-positive, histological grade I. DNA diploid with DI 1.02. CV = 1.2%. (**d**) From cancer, belonging to both the cancers with predominance of DCIS and the screening group (groups I + IV), tumour size 10 mm, node-negative, histological grade I. DNA aneuploid and heterogeneous with three hyperdiploid clones with DIs 1.11, 1.23 and 1.33. CV = 3.0%. The insert shows the diploid/hyperdiploid region from channel 175 to 280. (**e**) From node-negative cancer (group III), tumour size 22 mm, node-negative, histological grade I. DNA hypodiploid with DI 0.97, CV = 1.2%. Although the DI is in the diploid range, the sample is classified as DNA hypodiploid due to the coincidence with a second peak with DI. 1.01. The insert shows the hypodiploid/diploid/hyperdiploid region from channel 175 to 230. (**f**) From small clinical cancer (group II), tumour size 8 mm, node-negative, invasive lobular carcinoma. DNA aneuploid with two hyperdiploid clones with DIs 1.06 and 1.10, CV = 1.4%. The insert shows the diploid/hyperdiploid region from channel 185 to 240.

clones, as supported also by the biological findings. Thus, the DNA hyperdiploid clones are in particular present in histological grade I tumours, similar to the findings in the carcinomas with predominance of DCIS. The slight DNA hyperploidy found by FCM may correspond to some of the subsets of breast carcinomas that by chromosomal analysis have been found to be characterised by only minimal deviations from the normal 46,XX karyotype (Pandis *et al.*, 1995). Examples of hyperdiploid clones are given in Figure 3a, d and f.

Intratumoral DNA heterogeneity showed agreement between the clinical groups, with frequencies of 35% and 42%, compared with 33% in carcinomas with predominance of DCIS and 37% in the series of pure DCIS (Ottesen *et al.*, 1995*a*). In the Danish study of 421 node-negative carcinomas, with a median tumour size of 25 mm (Balslev *et al.*, 1994), a frequency of 24% was found. This study is comparable with ours, because unfixed, frozen tissue samples were prepared and measured by flow cytometry in the same way, and the data were interpreted by the same statistician (IJC). Results from previous studies raised the possibility that heterogeneity might be less in tumours from patients with early cancers (Beerman *et al.*, 1991). Our results indicate that heterogeneity is present with the same frequency in carcinomas of smaller size and even in the preinvasive stage of DCIS.

The second sample analysed in 56 cases from groups III and IV did not influence the DNA ploidy pattern to a great extent. Although a bias in the estimation of the frequency of DNA ploidy is present, this does not in any way invalidate our results. The reason for these differences could in most cases be intratumoral DNA heterogeneity. In some cases, the probably explanation is a lower CV in the second sample. The exclusion of the second sample would have resulted in a larger difference between the clinical and the screening groups.

We found that DNA diploid clones had a significantly lower SPF in comparison with DNA aneuploid clones. This is in agreement with results from the literature (Kallioniemi *et al.*, 1988*b*; Hatschek *et al.*, 1989; Ferno *et al.*, 1992; Stål *et al.*, 1992; Balslev *et al.*, 1994). Like the other DNA parameters, the SPF values of the IC groups are similar to those of DCIS lesions (Ottesen *et al.*, 1995*a*). The figure of 5% in the DNA diploid ICs is similar to that found in benign samples included in the DCIS study. This indicates a very low growth rate for these tumours. We were able to demonstrate a significant difference in SPF between the screening group and the small clinical cancers, however, only for the DNA aneuploid subpopulations. In comparison, Kallioniemi *et al.* (1988*b*) found lower SPF in the screening group compared with the control group, for the DNA diploid as well as the DNA aneuploid subpopulations, whereas Hatschek (1989) did not find any differences.

The histopathological classification demonstrated a lack of relationship between traditional parameters in the present series of carcinomas. No association could be proven between lymph node status and tumour size or histological grade. With respect to DNA ploidy, an association was shown between lymph node status and DNA diploidy/aneuploidy and also between histological grade and subclasses of DI, whereas DI was not correlated with tumour size.

In conclusion, the present study shows

(1) that the DNA ploidy data of the selected group of ductal carcinomas with predominance of DCIS were similar to those of the two clinical groups, i.e. the small clinical cancers and the node-negative cancers; and
(2) that they differed significantly from those of the screening-detected cancers.

These may represent a biologically specific group of low-malignant, slowly growing tumours. In addition, the DNA findings in the clinical cancers, as regards both DNA ploidy, DI distribution, frequency of intratumoral heterogeneity and SPF, are in agreement with the findings in node-negative breast cancers of larger size, as well as in preinvasive DCIS lesions. The only difference to the DCIS lesions was the additional presence, particularly in highly differentiated cancers, of DNA near-diploid clones that may possibly be related to invasion.

**Acknowledgement**
This study was supported by the Danish Cancer Society (76-010).

**References**

ALANEN KA, KLEMI PJ, JOENSUU H, KUJARI H AND PEKKALA E. (1989). Comparison of fresh, ethanol-preserved, and paraffin-embedded samples in DNA flow cytometry. *Cytometry*, **10**, 81–85.

BALSLEV I, CHRISTENSEN IJ, RASMUSSEN BB, LARSEN JK, LYKKESFELDT AE, THORPE SM, ROSE C, BRIAND P AND MOURIDSEN HT. (1994). Flow cytometric DNA ploidy defines patients with poor prognosis in node-negative breast cancer. *Int. J. Cancer*, **56**, 16–25.

BEERMAN H, KLUIN PhM, HERMANS J, VAN DE VELDE CJH AND CORNELISSE CJ. (1990). Prognostic significance of DNA ploidy in a series of 690 primary breast cancer patients. *Int. J. Cancer*, **45**, 34–39.

BEERMAN H, SMIT VTHBM, KLUIN PhM, BONSING BA, HERMANS J AND CORNELISSE CJ. (1991). Flow cytometric analysis of DNA stemline heterogeneity in primary and metastatic breast cancer. *Cytometry*, **12**, 147–154.

CORNELISSE JC, TANKE HJ, DE KONING H AND BRUTEL DE LA RIVIERE G. (1983). DNA ploidy analysis and cytologic examination of sorted cell populations from human breast tumors. *Anal. Quant. Cytol.*, **5**, 173–183.

ERIKSSON ET, SCHIMMELPENNING H, SVANE G, AZAVEDO E AND AUER GU. (1994). Prognostic indicators in small ( $\leqslant$ 10 mm), mammographically detected invasive mammary adenocarcinomas. In: *Biological Markers in Diagnostic Pathology of Benign, Pre-malignant and Malignant Breast Disease*, paper VI (thesis). Karolinska Institute: Stockholm.

EWERS S-B, BALDETORP B, KILLANDER D AND LÅNGSTRØM E. (1989). Flow cytometry DNA ploidy and number of cell populations in the primary breast cancer and their correlation to the prognosis. *Acta Oncol.*, **28**, 913–918.

FERNO M, BALDETORP B, BORG Å, OLSSON H, SIGURDSSON H AND KILLANDER D. (1992). Flow cytometric DNA Index and S-phase fraction in breast cancer in relation to other prognostic variables and to clinical outcome. *Acta Oncol.*, **31**, 157–165.

FRIERSON HF. (1988). Flow cytometric analysis of ploidy in solid neoplasms: comparison of fresh tissues with formalin-fixed paraffin-embedded specimens. *Hum. Pathol.*, **19**, 290–294.

HATSCHEK T, FAGERBERG G, STÅL O, SULLIVAN S, CARSTENSEN J, GRØNTOFT O AND NORDENSKJØLD B. (1989). Cytometric characterization and clinical course of breast cancer diagnosed in a population-based screening program. *Cancer*, **64**, 1074–1081.

HEDLEY DW. (1993). DNA flow cytometry and breast cancer. *Breast Cancer Res. Treat.*, **28**, 51–53.

KALLIONIEMI OP. (1988*a*). Comparison of fresh and paraffin-embedded tissue as starting material for DNA flow cytometry and evaluation of intratumor heterogeneity. *Cytometry*, **9**, 164–169.

KALLIONIEMI OP, KARKKAINEN A, AUVINEN O, MATTILA J, KOIVULA T AND HAKAMA M. (1988*b*). DNA flow cytometric analysis indicates that many breast cancers detected in the first round of mammographic screening have a low malignant potential. *Int. J. Cancer*, **42**, 697–702.

OTTESEN GL, CHRISTENSEN IJ, LARSEN JK, CHRISTIANSEN J, HANSEN B AND ANDERSEN J. (1995*a*). DNA analysis of *in situ* ductal carcinoma of the breast by flow cytometry. *Cytometry; Comm. Clin. Cytometry; Comm. Clin. Cytometry*, **22**, 168–176.

OTTESEN GL, CHRISTENSEN IJ, LARSEN JK, HANSEN B AND ANDERSEN JA. (1995*b*). Flow cytometric DNA analysis of breast cancers with predominance of carcinoma in situ. A comparison of the premalignant and malignant component. *Clin. Cancer Res.* **1**, 881–888.

OTTESEN GL, CHRISTENSEN IJ, LARSEN JK, HANSEN B AND ANDERSEN JA. (1995c). Tissue disaggregation for flow cytometric DNA analysis: A comparison of five-needle aspiration and an automated mechanical procedure. *Cytometry; Comm. Clin. Cytometry*. (submitted).

PANDIS N, JIN Y, GORUNOVA L, PETERSSON C, BARDI G, IDVALL I, JOHANSSON B, INGVAR C, MANDAHL N, MITELMAN F AND HEIM S. (1995). Chromosome analysis of 97 primary breast carcinomas: identification of eight karyotypic subgroups. *Genes Chrom. Cancer*, **12**, 173–185.

STÅL O, BRISFORS A, CARSTENSEN J, FERRAUD L, HATSCHEK T AND NORDENSKJØLD B. (1992). Interrelations between cellular DNA content, S-phase fraction, hormone receptor status and age in primary breast cancer. *Acta Oncol.*, **31**, 283–292.

UYTERLINDE AM, BAAK JPA, SCHIPPER NW, PETERSE HJ, MEIJER JWR, VOOYS PG AND MATZE E. (1991). Prognostic value of morphometry and DNA flow-cytometry features of invasive breast cancers detected by population screening: comparison with control group of hospital patients. *Int. J. Cancer*, **48**, 173–181.

VINDELØV LL, CHRISTENSEN IJ AND NISSEN NI. (1983). A detergent-trypsin method for the preparation of nuclei for flow cytometric DNA analysis. *Cytometry*, **3**, 323–327.

VINDELØV LL AND CHRISTENSEN IJ. (1990). A review of techniques and results obtained in one laboratory by an integrated system of methods designed for routine clinical flow cytometric DNA analysis. *Cytometry*, **11**, 753–770.

WHO. (1968). Histological typing of breast tumours, 1st edn. WHO; Geneva.

WHO. (1981). Histological typing of breast tumours, 2nd edn. WHO; Geneva.

ZALUPSKI MM, MACIOROWSKI Z, RYAN JR, ENSLEY JF, HUSSEIN ME, SUNDARESON AS AND BAKER LH. (1993). DNA content parameters of paraffin-embedded soft tissue sarcomas: optimization of retrieval technique and comparison to fresh tissue. *Cytometry*, **14**, 327–333.